# Key role of gut microbiota in anhedonia-like phenotype in rodents with neuropathic pain

**DOI:** 10.1038/s41398-019-0379-8

**Published:** 2019-01-31

**Authors:** Chun Yang, Xi Fang, Gaofeng Zhan, Niannian Huang, Shan Li, Jiangjiang Bi, Riyue Jiang, Ling Yang, Liying Miao, Bin Zhu, Ailin Luo, Kenji Hashimoto

**Affiliations:** 1 Department of Anesthesiology, Tongji Hospital, Tongji Medical College, Huazhong University of Science and Technology, Wuhan, China; 2 Department of Internal Medicine, The Third Affiliated Hospital of Soochow University, Changzhou, China; 3 Division of Clinical Neuroscience, Chiba University Center for Forensic Mental Health, Chiba, Japan

## Abstract

Patients with chronic neuropathic pain frequently suffer from symptoms of anhedonia, which is a core symptom of depression. Accumulating studies suggest that gut microbiota may play a role in depression via gut–microbiota–brain axis. However, it is unknown whether gut microbiota plays a role in neuropathic pain-associated anhedonia. Here, we used a rat model of spared nerve injury (SNI). Hierarchical cluster analysis of sucrose preference test (SPT) results was used to classify the SNI rats with or without anhedonia-like phenotype. The 16S ribosomal RNA sequencing analysis showed abnormal composition of gut microbiota in the anhedonia susceptible compared to sham-operated rats and resilient rats. Furthermore, antibiotics-treated mice showed pain as well as depression-like and anhedonia-like phenotypes, suggesting a role of gut microbiota in these abnormal behaviors. Transplantation of fecal microbiota from anhedonia susceptible rats into antibiotics-treated pseudo-germ-free mice significantly exaggerated pain and depression-like phenotypes, including anhedonia. In contrast, transplantation of fecal microbiota from resilient rats into antibiotics-treated pseudo-germ-free mice significantly improved pain and depression-like phenotypes, including anhedonia. In conclusion, this study suggests that abnormal composition of gut microbiota may contribute to anhedonia susceptibility post SNI surgery, and that gut microbiota also plays a role in the pain as well as depression-like phenotypes. Interestingly, fecal microbiota transplantation from SNI rats with or without anhedonia can alter pain, depression-like and anhedonia-like phenotypes in the pseudo-germ-free mice. Therefore, it is likely that gut microbiota plays a key role in the pain as well as depression-like phenotypes including anhedonia in rodents with neuropathic pain.

## Introduction

Pain is one of the most common ailments that make patients seek medical treatment, representing a major clinical, social, and economic problem. Patients with chronic pain often suffer with depressive symptoms. Clinical studies have demonstrated that the incidence of comorbid chronic pain and depression is ~30–50%^1–3^. Furthermore, a longitudinal study showed that chronic pain strongly predicts the development of more depressive symptoms in patients with pain compared with patients without pain^4^. Thus, it is likely that individual differences exist in the development of pain and depression comorbidities^1,5^. Collectively, comorbid pain and depression are a serious clinical, social, and economic issue that needs to be resolved, although the underlying mechanisms and therapeutic strategies for managing this comorbidity remain undetermined. In addition, the possible predisposing factors for individual differences in these comorbidities remain poorly understood.

The gut–microbiota–brain axis is a complex multi-organ bidirectional signaling system between the microbiota and brain that plays a fundamental role in host physiology, homeostasis, development, and metabolism^6–10^. Furthermore, the gut microbiota is reported to play a role in the visceral pain or chemotherapy-induced pain^11,12^, suggesting a possible role of the gut microbiota in visceral pain^13–15^. Accumulating studies suggest that the gut microbiota may contribute to the pathogenesis of depression and the antidepressant actions of certain compounds^6,7,16–23^. However, it is currently unknown how the gut microbiota plays a role in the depressive symptoms in patients with neuropathic pain.

According to the Diagnostic and Statistical Manual of Mental Disorders (DSM-5), the two core symptoms of depression are depressed mood and anhedonia (loss of pleasure). It is, therefore, important to study the role of depressive symptoms including anhedonia in pain because it specifically predicts worse treatment outcome^24^ and a longer and more severe course of depression^25^.

The purpose of the present study was to examine the role of the gut microbiota in pain and depression-like phenotypes including anhedonia in rodents following spared nerve injury (SNI) surgery. Furthermore, we investigated whether transplantation of fecal microbiota from rats with or without anhedonia into antibiotics-treated pseudo-germ-free mice could affect pain and depression-like phenotypes including anhedonia in these mice.

## Materials and methods

### Animals

Two-month-old male C57BL/6 mice and two-month-old Sprague Dawley rats were obtained from the Animal Center of Tongji Hospital. Animals were adapted to their environmental conditions for 7 days before experiments. Animals were housed in polypropylene cages with food and water ad libitum. The room temperature was maintained at 22 ± 2 °C and a relative humidity of 60% ± 5% on a 12-h light/dark cycle. All experimental protocols and animal handling procedures were carried out in strict accordance with the recommendations in the Guide for the Care and Use of Laboratory Animals, published by the National Institutes of Health (NIH Publications No. 80–23, revised in 1996). This study was approved by the Ethical Committee on Animal Experimentation of the Tongji Hospital, Tongji Medical College, Huazhong University of Science and Technology.

### Spared nerve injury (SNI)

The SNI surgery was performed as previously described^26–28^. Rats were anesthetized with 10% chloral hydrate (3 ml/kg) and then the skin of left thigh was incised. The sciatic nerve and its three terminal branches after bluntly dissecting biceps femoris muscle were totally exposed. The common peroneal and tibial nerves were ligated with a 4-0 silk and cut off the distal to the ligation. The muscle and skin were sutured with a 4-0 silk. Rats in the sham group were exposed to the sciatic nerve and its three terminal branches but without ligated and cut off the common peroneal and tibial nerves.

### Mechanical withdrawal test (MWT)

Before MWT, rats were placed in plexiglass chambers with a wire net floor for 30 min avoiding the stress resulting from the test conditions^26–28^. The Electronic Von Frey (UGO BASILE S.R.L., Italy) filaments were applied to the lateral 1/3 of right paws. The paws quick withdrawal or flinching was considered as a positive response. Every filament stimuli were applied four times with a period of 30 s interval.

### Tail-flick test (TFT)

The tail-flick latency was defined by the latency time to withdrawal or flick from the hot water (48 °C). The length of tail in hot water was about 1–1.5 cm. The latency time was measured with second chronograph. The tail quick withdrawal or flick was considered as a positive response and stop the second chronograph. Every mouse was applied three times with a period of 30-min interval^29^.

### Sucrose preference test (SPT)

Animals were exposed to water and 1% sucrose solution for 48 h, followed by 24 h of water and food deprivation and a 24-h exposure to two identical bottles, one is water, and another is 1% sucrose solution. The bottles containing water and sucrose were weighed before and at the end of this period and the sucrose preference was determined^26–28^.

### Locomotion

The locomotor activity was measured by an animal behavior analysis system YH-OF-M/R (Yihong Co., Ltd., Wuhan, China), the mice were placed in experimental cages (length × width × height: 1000 × 1000 × 450 mm) for 5 min. The behavioral data and trace were recorded. Cages were cleaned between testing session.

### Tail suspension test (TST)

A small piece of adhesive tape placed approximately 2 cm from the tip of the tail for mouse. The immobility time was recorded by camera for 10 min^21^. Mice were considered immobile only when they hung passively and completely motionless. The behavioral performance of mice in TST was analyzed by YH-TS behavioral analysis system (Yihong Co., Ltd., Wuhan, China).

### Forced swimming test (FST)

The FST was tested by an automated forced-swim apparatus YH-FST (Yihong Co., Ltd., Wuhan, China). The mice were placed individually in a cylinder (diameter: 25 cm; height: 35 cm) containing 20 cm of water, maintained at 23 ± 1 °C. The immobility time for mouse was recorded for 5 min.

### Pseudo germ-free mice modeling

Based on the our previous report^30^, broad-spectrum antibiotics (ampicillin 1 g/L, neomycin sulfate 1 g/L, metronidazole 1 g/L, Sigma-Aldrich Co. Ltd, MO, USA) dissolved in drinking water were given ad libitum to C57BL/6 mice for 14 consecutive days. The drinking solution was renewed every 2 days.

### Transplantation of fecal microbiota

Rats with or without anhedonia were divided after SNI surgery as described above. Subsequently, the rats were placed in a clean cage with sterilized filter paper. The feces samples were collected immediately after defecation in a sterilized centrifuge tube. The filter paper was replaced for different rat samples. Fecal samples were stored in a −80 °C freezer until analysis and transplantation. Fecal microbiota was prepared by diluting a 1 g fecal sample obtained from anhedonia susceptible rats or resilient rats in 10 mL of sterile PBS. The fecal material was suspended and 0.2 mL of the suspension was guided by gavage into each mouse recipient for consecutive 14 days^30^.

From day −7 to −1, mice were acclimated to new environment. On day −1, MWT and TFT baseline of mice were measured and recorded before drinking large doses of antibiotic solution and fecal transplantation. From day 0 to 14, large doses of antibiotics were administered. From day 14 to 28, fecal microbiota from the rats was transplanted to pseudo-germ-free mice. Subsequently, behavioral tests were measured from day 29 to 32. On day 34, fecal samples were collected for 16S rRNA gene sequencing analysis.

### 16S rRNA analysis of fecal samples

**Fig. 1:**
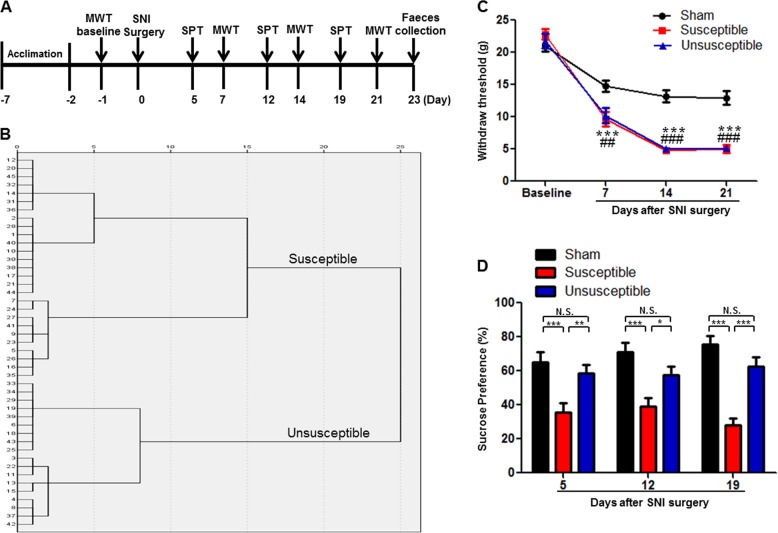
Comparisons of MWT and SPT among sham, anhedonia susceptible and resilient rats after SNI surgery. **a** The schedule of SNI and behavioral tests. SNI was performed 7 days after acclimation. MWT was measured on day 7, 14, and 21 after SNI surgery, respectively. SPT was performed on day 5, 12, and 19 after SNI surgery, respectively. Fecal samples were collected on day 23. **b** Dendrogram of hierarchical clustering analysis. A total of 45 SNI rats were divided into anhedonia susceptible and resilient groups by SPT results of hierarchical clustering analysis. **c** MWT (Time: *F*_3,21 = _209.152_,_ *P* < 0.001; Group: *F*_2,14 = _36.370_,_ *P* < 0.001; Interaction: *F*_6,42 = _10.159_,_ *P* < 0.001). ***P < 0.001, susceptible group v.s. sham group; ^##^*P* < 0.01 or ^###^ *P* < 0.001, resilient group v.s. sham group. **d** SPT (Time: *F*_2,14 = _3.125_,_ *P* = 0.456; Group: *F*_2,14 = _25.506_,_ *P* < 0.001; Interaction: *F*_4,28 = _1.274_,_ *P* = 0.304). *P < 0.05, **P < 0.01 or ***P < 0.001. Data are shown as mean ± S.E.M. (n = 8). N.S. not significant, SNI spared nerve injury, SPT sucrose preference test, WMT mechanical withdrawal test

The fecal samples were collected after behavioral tests (Fig. 1a). Samples were placed in 1.5 ml tubes, snap-frozen on dry ice, and stored at −80 °C. The 16 S rRNA analysis of the fecal samples was performed at OEbiotech Co., Ltd. (Shanghai, China). DNA extraction was performed using TIANamp stool DNA kits (Tiangen Biotechnology Company, Beijing, China). Genomic DNA was amplified in 50 μL triplicate reactions with bacterial 16S rRNA gene (V3-V4 region)-specific primers: 338 F (5′-ACTCCTACGGGAGGCAGC-3′) and 806R (5′-GG ACTACHVGGGTWTCTAAT-3′). The reverse primer contained a sample barcode and both primers were connected with an Illumina sequencing adapter. PCR products were purified and the concentrations adjusted for sequencing on an Illumina Miseq PE300 system. Original sequencing reads from the sample were sorted by unique barcodes, followed by the removal of the barcode, linker, and PCR primer sequences. The resultant sequences were screened for quality and 70 or more base pairs were selected for bioinformatics analysis. All sequences were classified using the NCBI BLAST and SILVA databases. Distance calculation, operational taxonomic units (OTU) cluster, rarefaction analysis, and estimator calculation (α-diversity and β-diversity) were performed by the MOTHUR program^30^.

### Statistical analysis

Values presented are expressed as mean ± standard error of the mean (S.E.M.). Statistical analyzes were performed using SPSS software version 17.0 (SPSS Inc., Armonk, New York, USA). In Hierarchical cluster analysis, the data were firstly standardized by *z* scores. Then, hierarchical cluster analysis of SPT results was performed using Ward’s method and applying squared Euclidean distance as the distance measure, and mice were classified as anhedonia susceptible rats or anhedonia unsusceptible (or resilient) rats^24–26^. Behavioral tests were analyzed by one-way or two-way analysis of variance (ANOVA), followed by post hoc Tukey’s test. Other data were analyzed by one-way ANOVA followed by post-hoc Tukey’s test, or Fisher’s exact test. *P*-values < 0.05 were considered statistically significant.

## Results

### Comparisons of MWT and SPT among sham, anhedonia susceptible, and resilient rats

A total of 45 rats were scheduled to undergo SNI surgery for mimicking neuropathic pain (Fig. 1a). SNI rats were divided into anhedonia susceptible and resilient rats by the results of by hierarchical cluster analysis of SPT (Fig. 1b). Body weights in the anhedonia susceptible rats and resilient rats at day 22 were significantly lower than that of sham-operated rats (Supplemental Figure S1). The results of MWT were significantly decreased in both anhedonia susceptible and resilient rats on day 7, 14, and 21 after SNI surgery (Fig. 1c). Interestingly, SPT, a test for anhedonia symptom in rodents, was significantly decreased in anhedonia susceptible rats, but not in the resilient rats, on day 5, 12, and 19 after SNI surgery (Fig. 1d). The data are consistent with our previous reports^26–28^.

### Comparisons of differential profiles in gut microbiota among sham, anhedonia susceptible and resilient rats

**Fig. 2:**
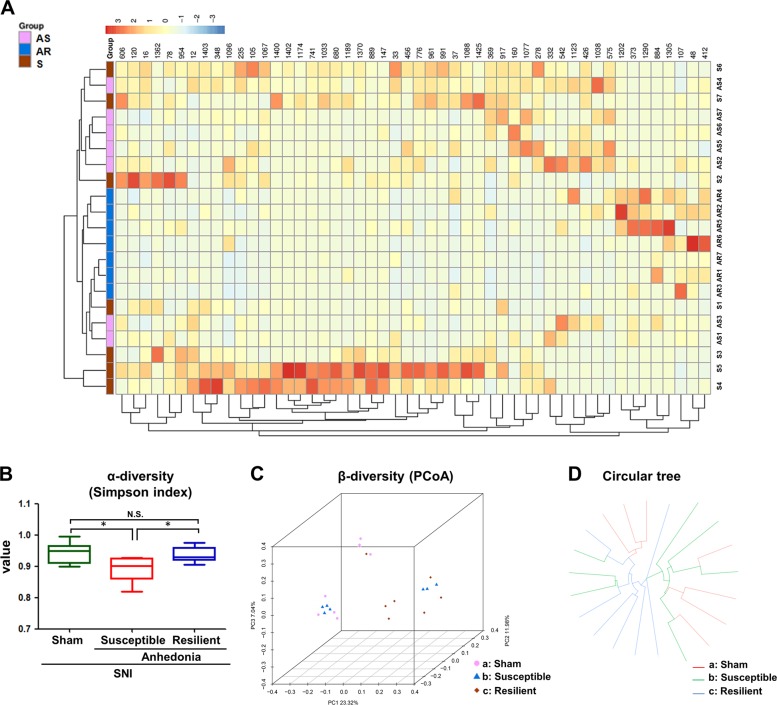
Differential profiles of gut microbiota among sham, anhedonia susceptible and resilient rats after SNI. **a** Heat map of different levels among the groups. **b** Simpson index (*F*_2, 18 = _5.098_,_ *P* = 0.018), an α-diversity indicator, among the groups. **c** Dendrogram of a β-diversity indicator PCoA. **d** Dendrogram of circular tree. α-Diversity data are shown as mean ± S.E.M. (*n* = 7). *P < 0.05. AS anhedonia susceptible, AR anhedonia resilient, N.S. not significant, PCoA principal coordinates analysis, S sham

We used 16S rRNA gene sequencing to determine alterations in the gut microbiota composition among the three groups. A large number of gut bacteria were altered among the three groups (Fig. 2a). α-Diversity refers to the diversity of bacteria or species within a community or habitat. Anhedonia susceptible rats showed a significant decrease in α-diversity value as compared with sham-operated rats or resilient rats (Fig. 2b). PCoA could present visual coordinates for the similarity or difference of data. It is a non-binding data analysis method that can be used to study the similarity or dissimilarity of sample community composition. In three-dimensional data of PCoA, the dots of sham group were far away from resilient group. It is noted that the four dots of anhedonia susceptible group were close to sham group, while the other three dots were close to resilient group (Fig. 2c). Circular tree data also showed that the composition of gut microbiota was quite different among the three groups (Fig. 2d).

### Differential levels of gut microbiota among sham, anhedonia susceptible, and resilient rats

At the phylum level, *Parcubacteria* was significantly increased in anhedonia susceptible rats compared to sham-operated rats, while *Parcubacteria* were not detected in resilient rats (Supplemental Figure S2A). Furthermore, *Verrucomicrobia* were also not detected in fecal samples of anhedonia susceptible and resilient rats (Supplemental Figure S2B). At the class level, anhedonia susceptible rats had lower levels of *Coriobacteriia* compared to sham-operated rats (Supplemental Figure S2C). Other bacteria at the class level that probably could not be currently nominated were significantly changed among the three groups (Supplemental Figure S2D).

At the order level, *Coriobacteriales*, other bacteria and *Parcubacteria* were significant different among the three groups (Supplemental Figure S2E~S2G). At the family level, *Bacteroidaceae*, *Porphyromonadaceae, Fusobacteriales other,* and *Parcubacteria other* were significantly increased in anhedonia susceptible group than those in sham group or resilient group (Supplemental Figure S2I~S2L). *Coriobacteriaceae* and *Vibrionaceae* were significantly changed among the three groups (Supplemental Figure S2H and S2M).

At the genus level, *Rothia*, *Bacteroides*, *Butyricimonas*, *Parabacteroides*, *Prevotellaceae UCG 001*, *Fusobacteriales other*, *Parcubacteria other*, *MNG7 other*, *Bilophila*, and *Aggregatibacter* were significantly increased in anhedonia susceptible group as compared with sham group or resilient group (Supplemental Figure S2N, S2P~S2S, S2X~S2AB). In contrast, *Enterorhabdus*, *XIII UCG 001*, *Acetatifactor*, *Lachnospiraceae UCG 005*, *Erysipelatoclostridium* and *Vibrionaceae other* were significantly decreased in anhedonia susceptible group compared to sham group or resilient group (Supplemental Figure S2O, S2T~S2W, S2AC).

At the species level, *Rothia Ambiguous taxa*, *Rothia other*, *Uncultured bacteriu*m, *Bacteroides ambiguous taxa*, *Butyricimonas other*, *Parabacteroides other*, *Prevotellaceae UCG 001*, *Family XIII UCG 001*, *Acetatifactor uncultured bacterium*, *Lachnospiraceae UCG 001*, *Lachnospiraceae UCG 005*, *UCG 010 other*, *Roseburia uncultured bacterium*, *Roseburia uncultured organism*, *Lachnospiraceae uncultured unidentified*, *Ruminiclostridium 5-uncultured Bacterium*, *Erysipelatoclostridium other*, *Fusobacteriales other, Parcubacteria other*, *MNG7 other*, *Aggregatibacter other*, *Vibrionaceae other* were significant altered among the three groups (Supplemental Figure S2AD~S2AY).

### Transplantation of fecal microbiota from rats with or without anhedonia into antibiotics-induced pseudo-germ-free mice

**Fig. 3:**
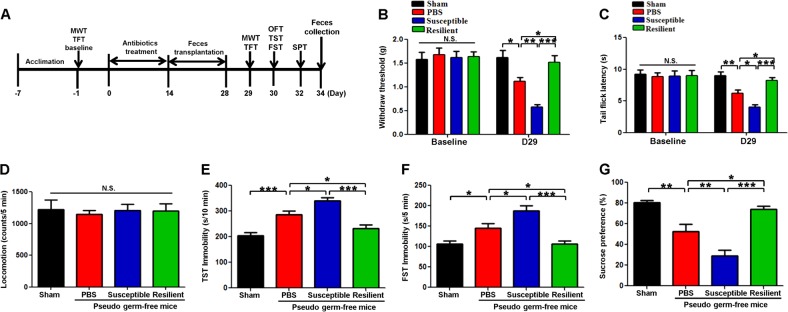
Effects of fecal microbiota transplantation from rats with or without anhedonia on behaviors in pseudo-germ-free mice. **a** Schedule of fecal microbiota transplantation on behavioral tests in pseudo-germ-free mice. From day −7 to day −1, mice were acclimated to new environment. On day −1 (baseline), MWT and TFT were measured. Then, mice were treated by drinking large doses of antibiotic solution for 14 consecutive days (day 0- day 14). Subsequently, mice were orally treated with fecal microbiota from rats with or without anhedonia from day 14 to day 28. MWT and TFT, OFT, TST and FST were performed on day 29 and 30, respectively. The SPT was performed on day 34. On day 34, fecal samples were collected for 16S rRNA gene sequencing testing. **b** MWT at day −1 (baseline). (Time: *F*_1,9 = _25.565_,_ *P* < 0.001; Group: *F*_3,27 = _7.433_,_ *P* < 0.001; Interaction: *F*_3,27 = _7.666_,_ *P* < 0.001). **c** TFT latency at day −1 (baseline). (Time: *F*_1,9 = _43.493_,_ *P* < 0.001; Group: *F*_3,27 = _6.619_,_ *P* = 0.002; Interaction: *F*_3,27 = _5.485_,_ *P* = 0.004). **d** locomotion at day 30 (*F*_3,36 = _0.093_,_ *P* = 0.964). **e** TST immobility at day 30 (*F*_3,36 = _23.978_,_ *P* < 0.001). **f** FST immobility (*F*_3,36 = _15.012_,_ *P* < 0.001). **g** SPT at day 32 (*F*_3,36 = _23.633_,_ *P* < 0.001). *P < 0.05, **P < 0.01 or ***P < 0.001. Data are shown as mean ± S.E.M. (*n* = 10). FST forced swimming test, N.S. not significant, OFT open-field test, SNI spared nerve injury, SPT sucrose preference test, TFT tail flick test, TST tail suspension test, WMT mechanical withdrawal test

Fourteen days after oral intake of antibiotics, fecal microbiota from rats with or without anhedonia were transplanted for consecutive 14 days into antibiotics-induced pseudo-germ-free mice (Fig. 3a). Changes in the body weights of the four groups during the experimental period were not different (Supplemental Figure S3). 1 day before antibiotics treatment, there were no changes in scores of MWT and latency of tail-flick test (TFT) among the all groups (Fig. 3b, c). Repeated administration of antibiotics significantly aggravated the scores of MWT, latency of TFT, and depression-like behaviors (e.g., immobility time of TST and FST, and sucrose preference). Furthermore, transplantation of fecal samples from rats with anhedonia significantly aggravated these behavioral abnormalities in these mice (Figure 3b~g). Interestingly, transplantation of fecal samples from resilient rat significantly restored these all behavioral performances (Figure 3b~g). These findings suggest that transplantation of fecal samples from rats with or without anhedonia significantly alter behavioral abnormalities in pseudo-germ-free mice.

### Effects of gut microbiota transplant on α-diversity and β-diversity in host fecal samples

**Fig. 4:**
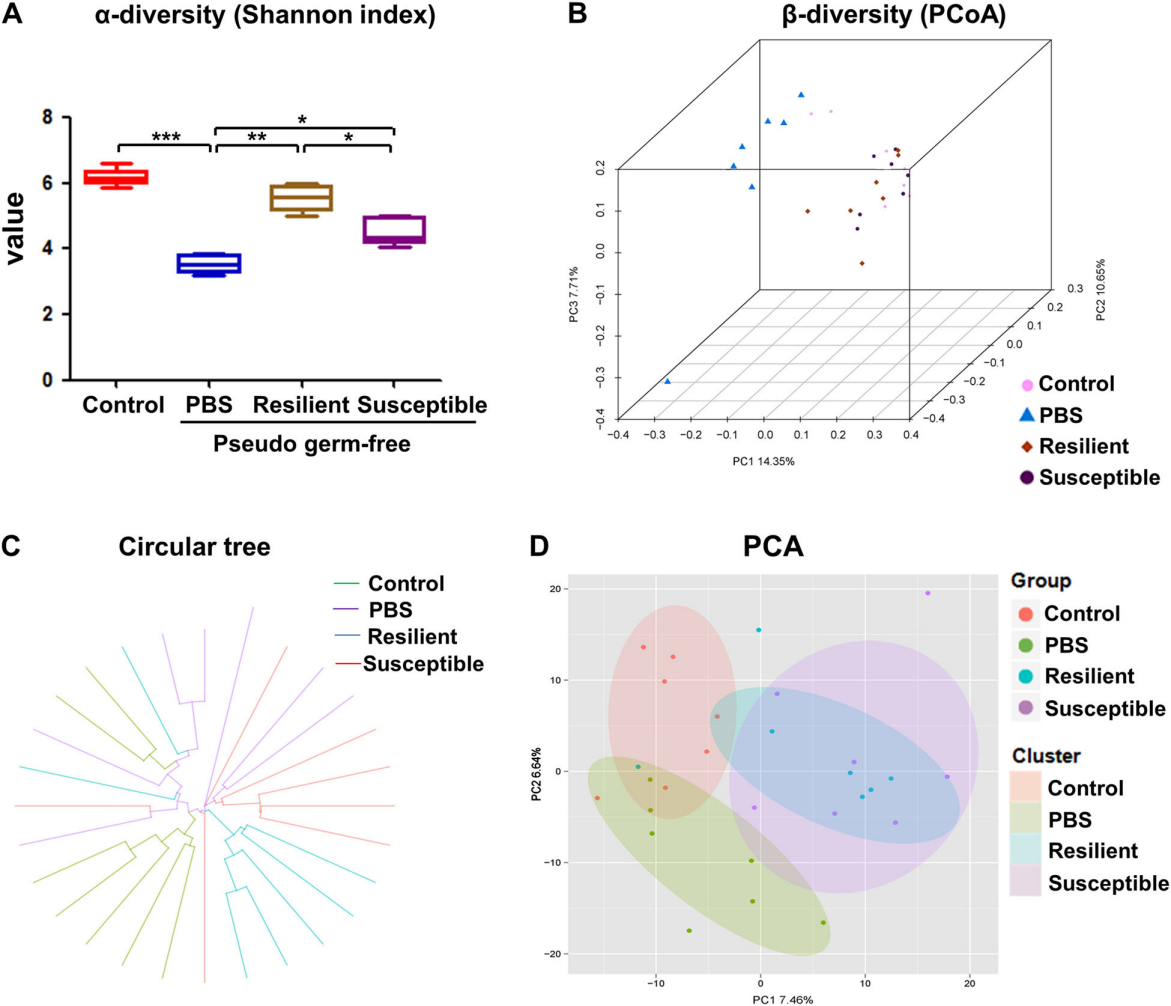
Differential profiles of gut microbiota transplant in pseudo-germ-free mice. **a** α-diversity Simpson index (*F*_3,24 = _89.807_,_ *P* < 0.001). **b** Dendrogram of β-diversity PCoA. **c** Dendrogram of circular tree. **d** Dendrogram of PCA. *P < 0.05, **P < 0.01 or ***P < 0.001. α-Diversity data are shown as mean ± S.E.M. (*n* = 7). PCA principal component analysis, PCoA principal coordinates analysis

Shannon index is one of the indicators for assessing the diversity of the microbiota. α-Diversity of Shannon index showed a significant decrease in PBS-treated pseudo-germ-free mice. However, both anhedonia susceptible and resilient groups significantly restored the decrease of Shannon index compared to PBS-treated group. Interestingly, there was a significant change in Shannon index between anhedonia susceptible group and resilient group (Fig. 4a). β-Diversity using PCoA demonstrated that the dots of control group were far away from the dots of PBS-treated group. In addition, the dots of anhedonia susceptible and resilient groups were also far away from the PBS-treated group, suggesting that the composition of gut microbiota of anhedonia susceptible and resilient groups were different from those in PBS-treated group. Compared with resilient group, the composition of gut microbiota in anhedonia susceptible group showed a different profile (Fig. 4b and Supplemental Figure S4). Furthermore, both circular tree and PCA showed that the composition of gut microbiota in the four groups were distinct (Fig. 4c, d).

### Effects of fecal microbiota transplantation on the levels of gut microbiota in host

**Fig. 5:**
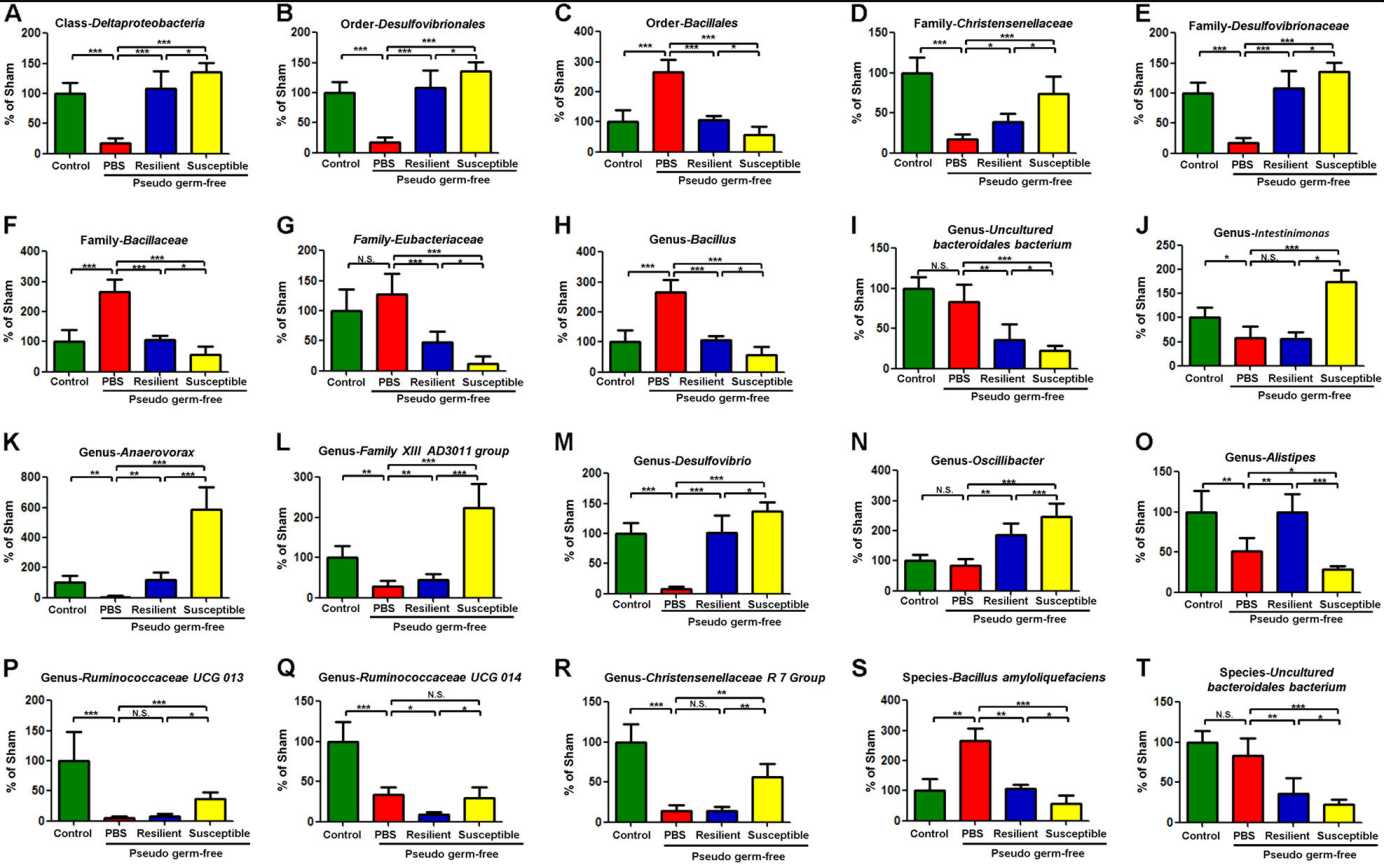
Effects of fecal bacteria transplant on levels of gut microbiota in pseudo-germ-free mice. **a** Class *Deltaproteobacteria* (*F*_3,24 = _3.774_,_ *P* = 0.024). **b** Order Desulfovibrionales (*F*_3,24 = _6.938_,_ *P* = 0.002). **c** Order *Bacillales* (*F*_3,24 = _8.093_,_ *P* < 0.001). **d** Family *Christensenellaceae* (*F*_3,24 = _5.289_,_ *P* = 0.006). **e** Family *Desulfovibrionaceae* (*F*_3,24 = _6.938_,_ *P* = 0.002). **f** Family *Bacillaceae* (*F*_3,24 = _8.093_,_ *P* < 0.001). **g** Family *Eubacteriaceae* (*F*_3,24 = _3.813_,_ *P* = 0.023). **h** Genus *Bacillus* (*F*_3,24 = _8.093_,_ *P* < 0.001). **i** Genus *Uncultured Bacteroidales Bacterium* (*F*_3,24 = _5.237_,_ *P* = 0.006). **j** Genus *Intestinimonas* (*F*_3,24 = _6.535_,_ *P* = 0.002). **k** Genus *Anaerovorax* (*F*_3,24 = _10.672_,_ *P* < 0.001). **l** Genus *Family XIII AD3011 group* (*F*_3,24 = _6.689_,_ *P* = 0.002). **m** Genus *Desulfovibrio* (*F*_3,24 = _8.468_,_ *P* < 0.001). **n** Genus *Oscillibacter* (*F*_3,24 = _5.292_,_ *P* = 0.006). **o** Genus *Alistipes* (*F*_3,24 = _3.345_,_ *P* = 0.036). **p** Genus *Ruminococcaceae UCG 013* (*F*_3,24 = _3.183_,_ *P* = 0.042). **q** Genus *Ruminococcaceae UCG 014* (*F*_3,24 = _7.01_,_ *P* = 0.002). **r** Genus *Christensenellaceae R 7 Group* (*F*_3,24 = _8.028_,_ *P* < 0.001). **s** Species *Bacillus Amyloliquefaciens* (*F*_3,24 = _8.093_,_ *P* < 0.001). **t** Species *Uncultured Bacteroidales Bacterium* (*F*_3, 18 = _8.121_,,_ *P* < 0.001). *P < 0.05, **P < 0.01 or ***P < 0.001. Data are shown as mean ± S.E.M. (*n* = 7). N.S. not significant

A total of 28 bacteria in the fecal samples were significantly altered among the four groups. Treatment with antibiotics significantly decreased the levels of *Deltaproteobacteria*, *Desulfovibrionales, Enterobacteriales, Christensenellaceae, Desulfovibrionaceae, Enterobacteriaceae, Prevotella 2, Ruminococcaceae NK4A214 group, Ruminococcaceae UCG 007, Intestinimonas, Anaerovorax, Family XIII AD3011 group, Desulfovibrio, Alistipes, Prevotellaceae NK3B31 group, Ruminococcaceae UCG 013, Ruminococcaceae UCG 014* and *Christensenellaceae R 7 Group* (Fig. 5a~b, d~e, j~m, o, p~r, and Supplemental S5A~F and S5H). In contrast, *Bacillales, Bacillaceae, Bacillus* and *Bacillus amyloliquefaciens* were significantly increased in the fecal samples of antibiotics-treated mice (Fig. 5c, f, h and Supplemental 5S). Additionally, there were significant differences in the levels of *Deltaproteobacteria, Desulfovibrionales, Bacillales, Christensenellaceae, Desulfovibrionaceae, Bacillaceae, Eubacteriaceae, Bacillus, Uncultured bacteroidales bacterium, Prevotella 2, Ruminococcaceae NK4A214 group, Ruminococcaceae UCG 007, Uncultured organism, Intestinimonas, Anaerovorax, Family XIII AD3011 group, Desulfovibrio, Oscillibacter, Alistipes, Prevotellaceae NK3B31 group, Ruminococcaceae UCG 013, Ruminococcaceae UCG 014, Christensenellaceae R 7 Group, Bacillus amyloliquefaciens, Uncultured bacteroidales bacterium* between the anhedonia susceptible and resilient groups (Fig. 5A~T, S5D~G and Supplemental S5H).

## Discussion

The present study demonstrated that although SNI rats suffered almost identical nociceptive damage, there were rats with or without anhedonia-like phenotypes. The 16S rRNA analysis showed abnormal composition of gut microbiota in the rats with or without anhedonia compared to sham-operated rats, suggesting a role of gut microbiota in the individual differences of anhedonia-like phenotype. Furthermore, antibiotics-treated pseudo-germ-free mice (>90% reduction of gut microbiota) showed depression-like and anhedonia-like phenotype compared to control group, suggesting a role of gut microbiota in behavioral abnormalities in these mice. Furthermore, transplantation of fecal microbiota from rats with anhedonia-like phenotype aggravated pain, depression-like and anhedonia-like phenotypes in the antibiotics-treated pseudo-germ-free mice. In contrast, transplantation of fecal microbiota from rats without anhedonia improved pain, depression-like and anhedonia-like phenotype in the antibiotic-treated pseudo-germ-free mice. Taken all together, these results suggest that abnormal composition of the gut microbiota plays a key role in the depression-like and anhedonia-like phenotypes in rodents with neuropathic pain. To the best of our knowledge, this is the first study demonstrating the role of gut microbiota in individual differences of anhedonia-like phenotype in the rats with neuropathic pain after SNI surgery. In addition, this is also the first study to establish the role of gut micobiota in the comorbidity of neuropathic pain and depression-like phenotypes including anhedonia in rodents.

Depression-like behaviors of hyperalgesic rats were highly diverse. Using resident-intruder social interaction and sleep–wake analyzes, chronic constriction injury induced a subgroup (~30%) of rats with altered dominant behavior^31^ and sleep–wake cycle^32^. Using the hierarchical cluster analysis, we divided SNI rats into two clusters: one group (~60%; anhedonia-like phenotype) with reduced sucrose preference in the SPT and the other (~40%; without anhedonia-like phenotype) with sucrose preference similar to that in sham-operated rats, consistent with our previous reports^26–28^. In this study, we also found that rats, regardless of the presence or abscence of anehedonia-like phenotype, exhibited similar MWT scores, suggesting that alterations in mood-related behaviors were independent on the degree of nociceptive damage, which was consistent with previous studies^26–28^.

The 16S rRNA analysis demonstrated abnormal composition of gut microbiota in the rats with or without anhedonia-like phenotype. In this study, we identified several microbiota which were altered in the rats with anhedonia-like phenotypes compared to sham-operated rats or resilient rats. At the genus level, *Butyricimonas, Parabacteroides, Prevotellaceae UCG 001, Bilophila* and *Aggregatibacter* were significantly increased in anhedonia susceptible group compared with sham group and resilient group. Very recently, we reported that (*R*)-ketamine, the rapid-acting antidepressant candidate, significantly attenuated the decrease in the levels of *Butyricimonas* in the susceptible mice after chronic social defeat stress^21^, suggesting that *Butyricimonas* may play a role in the (*R*)-ketamine’s antidepressant effects. In contrast, *XIII UCG 001*, and *Lachnospiraceae UCG 005* were significantly decreased in anhedonia susceptible group compared to sham group and resilient group. Collectively, these microbiota may play a role in anhedonia-like phenotype in mice although further study is needed.

At the species level, *Bacteroides ambiguous taxa*, *Butyricimonas other*, *Parabacteroides other*, *Prevotellaceae UCG 001*, *Family XIII UCG 001*, *Acetatifactor uncultured bacterium*, *Lachnospiraceae UCG 001*, and *Lachnospiraceae UCG 005* were significantly altered in the anhedonia susceptible rats compared to sham-operated rats or resilient rats. It seems that abnormal composition of these microbiota may play a role in anhedonia-like phenotype after SNI. In addition, it appears that alterations in the gut microbiota are independent on the degree of nociceptive damages following SNI. Overall, it is likely that abnormal composition of the gut microbiota might be associated with anhedonia-like phenotype in rats with neuropathic pain.

In this study, we found that antibiotics-treated mice showed pain as well as increased immobility time of TST and FST, decreased sucrose preference of SPT compared to control mice. It is, thus, likely that gut microbiota plays a role in pain as well as depression-like phenotype including anhedonia since more than 90% of the gut microbiota could be killed by antibiotics^33^. However, a previous report^16^ found that the immobility time of FST in the germ-free mice was significantly lower than that of specific pathogen-free (SPF) mice, suggesting that the absence of gut microbiota may cause resilience in the FST. In this study, the authors did not perform other behavioral tests, such as TST and SPT^16^. Although the reasons underlying this discrepancy are unknown, the use of mice (germ-free mice vs. pseudo-germ-free mice) and behavioral tests (FST vs. TST, FST, SPT) may contribute to the discrepancy. Nonetheless, further study is needed to confirm the role of gut microbiota in depression-like behaviors in rodents.

Interestingly, fecal microbiota transplantation from anhedonia susceptible rats into pseudo-germ-free mice significantly aggravated pain as well as depression-like and anhedonia-like phenotypes in these mice. In contrast, fecal microbiota transplantation from resistant rats significantly restored pain as well as depression-like and anhedonia-like phenotypes in the pseudo-germ-free mice. In this study, we found a number of microbiota which were altered after fecal microbiota transplantation from rats with or without anhedonia. Interestingly, we also found that fecal microbiota transplantation from resistant rats significantly restored the reduced levels of *Alistipes* in the pseudo-germ-free mice, suggesting that increase in *Alistipes* may play a role in the improvement of behavioral abnormalities. Depressed patients treated with antidepressants had increased levels of *Alistipes* compared to healthy subjects^6^. Additionally, one clade within *Alistipes* was significantly associated with depression^34^. Furthermore, Saulnier et al.^35^ reported that higher levels of *Alistipes* were associated with a greater frequency of abdominal pain in patients with irritable bowel syndrome, suggesting that *Alistipes* may be associated with gut inflammation. Nonetheless, further study is needed to clarify the role of *Alistipes* in medication-free depressed patients with neuropathic pain. Collectively, it is likely that the gut microbiota plays a role in these behavioral abnormalities in pseudo-germ-free mice although we did not identify the specific microbiota species that contribute to the development of depression-like and anhedonia-like phenotypes.

Zheng et al.^16^ reported that fecal microbiota transplantation of germ-free mice with ‘depression microbiota’ from depressed patients resulted in depression-like behaviors compared with colonization with ‘healthy microbiota’ from healthy control subjects. Significantly the altered phylum-level bacteria taxa of the ‘inputted’ human ‘depression microbiota’ (characterized by alterations in *Firmicutes, Actinobacteria* and *Bacteroidetes*) were efficiently captured in the ‘output’ mouse fecal communities of ‘humanized’ depressed mice. The study suggests that the alterations of gut microbiota and resulting induction of depression-like behaviors are transmissible^16^. If we can identify the specific microbiota that contribute to depression-like and anhedonia-like phenotypes in SNI rats, these microbiota would be novel therapeutic targets for depression and anhedonia in patients with neuropathic pain.

Our study has some limitations. First, we did not investigate the role of sex difference in gut microbiota composition in neuropathic pain-related anhedonia, although the previous studies showed a gender-dependent difference in the gut microbiota composition^36,37^. Further study on sex differences in neuropathic pain-induced anhedonia is needed. Second, brain-derived neurotrophic factor (BDNF) and its receptor TrkB signaling plays a critical role in depression, pain and other neuropsychiatric diseases^38–42^. Further studies are required to study whether gut microbiota improve neuropathic pain-related anhedonia through BDNF-TrkB signaling in the brain. Finally, we speculate that abnormal composition of gut microbiota may be shown in patients with neuropathic pain-related anhedonia. However, a large-scale clinical study is needed to investigate whether transplantation of gut microbiota from healthy control subjects could benefit in patients with neuropathic pain-related anhedonia.

In conclusion, the present study suggests that abnormal composition of gut microbiota may be associated with individual differences of anhedonia-like phenotype in rats with neuropathic pain. Furthermore, the fecal microbiota transplantation from SNI rats with or without anhedonia significantly can alter pain, depression-like and anhedonia-like phenotypes in the pseudo-germ-free mice. Therefore, it is likely that fecal microbiota transplantation from healthy control subjects would be a potential therapeutic approach for depression and anhedonia in patients with neuropathic pain.

## Supplementary information

Supplemental information
