# Supplementary material for: Key role of gut microbiota in anhedonia-like phenotype in rodents with neuropathic pain
Source: Transl Psychiatry. 2019 Jan 31;9:57. doi: 10.1038/s41398-019-0379-8 (PMC6355832; doi:10.1038/s41398-019-0379-8)
Supplement: Supplementary file 1 — Supplemental information [file 41398_2019_379_MOESM1_ESM.docx]

**Supplemental Information**

**
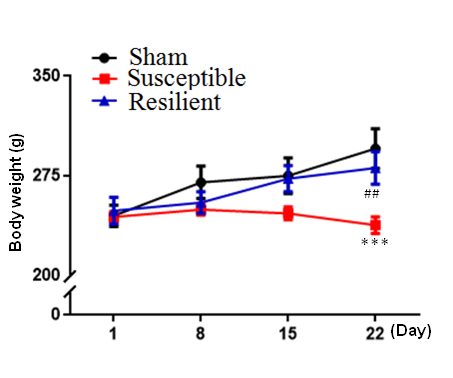
**

**Figure S1. Effects of SNI modeling and gut microbiota transplant on body weight in mice.** (A). Changes in body weight among sham, anhedonia susceptible and resilient groups (Time: F_3,21_ = 6.707_,_ *P* = 0.002; Group: F_2,14_ =2.177_,_ *P* = 0.150; Interaction: F_6,42_ = 3.085_,_ *P* = 0.014). ****P* < 0.001 v.s. sham, ^##^*P* < 0.01 v.s. susceptible. Data are shown as mean ± S.E.M. (n = 8 or 10).

**
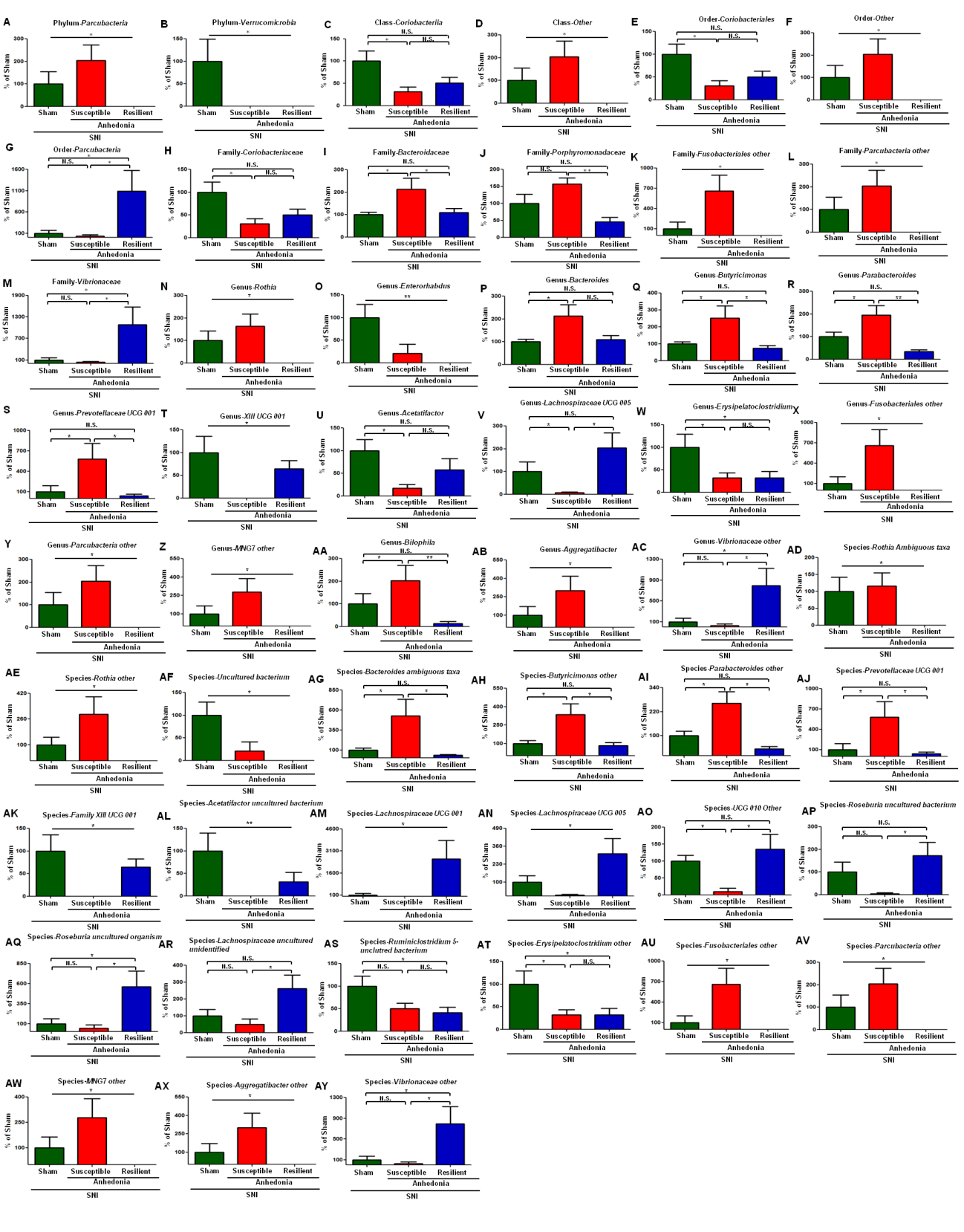
**

**Figure S2. Differential levels of gut bacteria among sham, anhedonia susceptible and resilient rats.** (A): Phylum *Parcubacteria* (Fisher’s exact test. *P* < 0.05). (B): Phylum *Verrucomicrobia* (Fisher’s exact test. *P* < 0.05). (C): Class *Coriobacteriia* (F_2,18_ =4.85_,_ *P* = 0.021). (D): Class *Other* (Fisher’s exact test. *P* < 0.05). (E): Order *Coriobacteriales* (F_2,18_ = 4.85_,_ *P* = 0.021). (F): Order *Other* (Fisher’s exact test. *P* < 0.05). (G): Order *Parcubacteria* (F_2,18_ = 4.028_,_ *P* = 0.036). (H): Family *Coriobacteriaceae* (F_2,18_ =4.85_,_ *P* = 0.021). (I) Family *Bacteroidaceae* (F_2,18_ = 4.16_,_ *P* = 0.033). (J): Family *Porphyromonadaceae* (F_2,18_ = 7.626_,_ *P* = 0.004). (K): Family *Fusobacteriales Other* (Fisher’s exact test. *P* < 0.05). (L): Family *Parcubacteria Other* (Fisher’s exact test. *P* < 0.05). (M): Family *Vibrionaceae* (F_2,18_ =4.340_,_ *P* = 0.029). (N): Genus *Rothia* (Fisher’s exact test. *P* < 0.05). (O): Genus *Enterorhabdus* (Fisher’s exact test. *P* < 0.01). (P): Genus *Bacteroides* (F_2,18_ =4.16_,_ *P* = 0.033). (Q): Genus *Butyricimonas* (F_2,18_ =5.027_,_ *P* = 0.018). (R): Genus *Parabacteroides* (F_2,18_ = 9.095_,_ *P* = 0.002). (S): Genus *Prevotellaceae UCG 001* (F_2,18_ =4.192_,_ *P* = 0.032). (T): Genus *XIII UCG 001* (Fisher’s exact test. *P* < 0.05). (U): Genus *Acetatifactor* (F_2,18_ =4.069_,_ *P* = 0.035). (V): Genus *Lachnospiraceae UCG 005* (F_2,18_ = 4.694_,_ *P* = 0.023). (W): Genus *Erysipelatoclostridium* (F_2,18_ = 3.919_,_ *P* =0.039). (X): Genus *Fusobacteriales Other* (Fisher’s exact test. *P* < 0.05). (Y): Genus *Parcubacteria Other* (Fisher’s exact test. *P* < 0.05). (Z): Genus *MNG7 Other* (Fisher’s exact test. *P* < 0.05). (AB): Genus *Aggregatibacter* (Fisher’s exact test. *P* < 0.05). (AC): Genus-*Vibrionaceae Other* (F_2 18_ = 4.739_,_ *P* = 0.022). (AD): Species *Rothia Ambiguous taxa* (Fisher’s exact test. *P* < 0.05). (AE): Species *Rothia Other* (Fisher’s exact test. *P* < 0.05). (AF): Species *Uncultured Bacterium* (Fisher’s exact test. *P* < 0.05). (AG): Species *Bacteroides Ambiguous taxa* (F_2,18_ =4.984_,_ *P* = 0.019). (AH): Species *Butyricimonas Other* (F_2, 18_ =6.679_,_ *P* = 0.007). (AI): Species *Parabacteroides Other* (F_2,18_ =10.416_,_ *P* = 0.001). (AJ) Species *Prevotellaceae UCG 001* (F_2,18_ =4.192_,_ *P* = 0.032). (AK): Species *Family XIII UCG 001* (Fisher’s exact test. *P* < 0.05). (AL): Species *Acetatifactor Uncultured Bacterium* (Fisher’s exact test. *P* < 0.01). (AM): Species *Lachnospiraceae UCG 001* (Fisher’s exact test. *P* < 0.05). (AN): Species *Lachnospiraceae UCG 005* (Fisher’s exact test. *P* < 0.01). (AO): Species-*UCG 010 Other* (F_2,18_ = 5.431_,_ *P* = 0.014). (AP): Species *Roseburia Uncultured Bacterium* (F_2,18_ =3.929_,_ *P* = 0.038). (AQ): Species *Roseburia Uncultured Organism* (F_2,18_ = 5.702_,_ *P* = 0.012). (AR): Species *Lachnospiraceae Uncultured Unidentified* (F_2,18_ =4.017_,_ *P* = 0.036). (AS): Species *Ruminiclostridium 5-unclutred Bacterium* (F_2,18_ =3,723_,_ *P* = 0.044). (AT): Species *Erysipelatoclostridium Other* (F_2, 18_ =3.919_,_ *P* = 0.039). (AU): Species *Fusobacteriales Other* (Fisher’s exact test. *P* < 0.05). (AV): Species *Parcubacteria Other* (Fisher’s exact test. *P* < 0.05). (AW): Species *MNG7 Other* (Fisher’s exact test. *P* < 0.05). (AX): Species *Aggregatibacter Other* (Fisher’s exact test. *P* < 0.05). (AY): Species *Vibrionaceae Other* (F_2,18_ =4.739_,_ *P* = 0.022). Data are shown as mean ± S.E.M. (n = 7). N.S.: not significant; SNI: spared nerve injury.

**
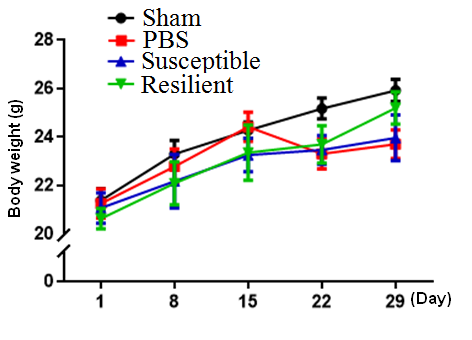
**

**Figure S3. Effects of SNI modeling and gut microbiota transplant on body weight in mice.** (A) Changes in body weight of mice receiving fecal microbiota transplantation from sham-operated, PBS-treated, anhedonia susceptible and resilient rats (Time: F_4,36_ = 28.228_,_ *P* < 0.001; Group: F_3,27_ =1.375_,_ *P* = 0.272; Interaction: F_12,108_ = 1.260_,_ *P* = 0.253). Data are shown as mean ± S.E.M. (n = 8 or 10).

**
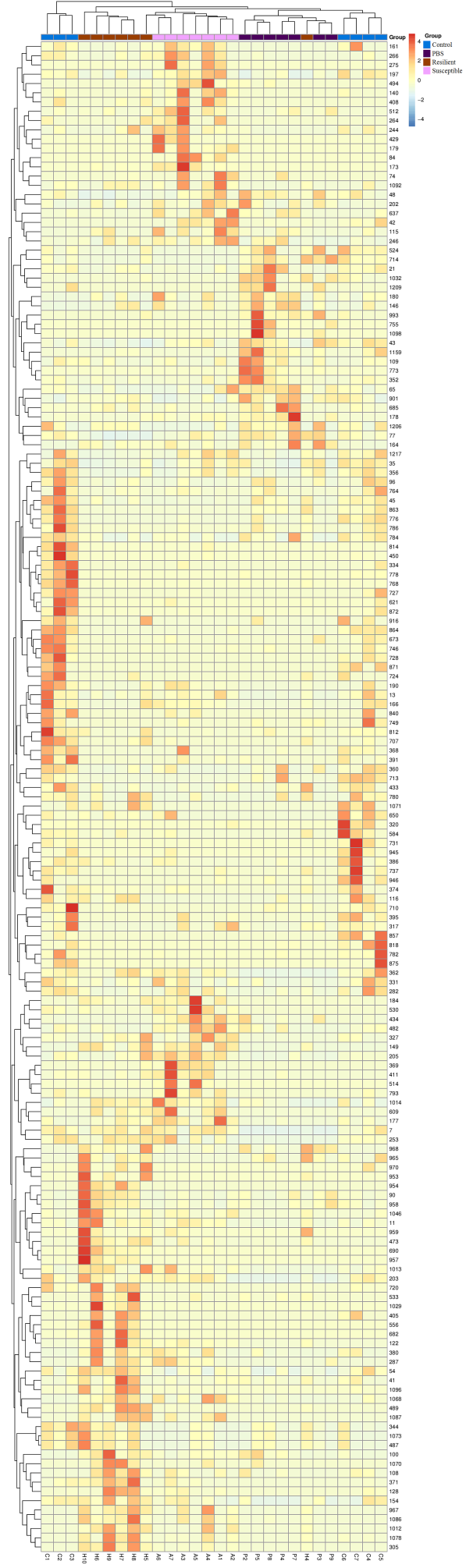
**

**Figure S4. A heat map of different levels among the four groups (Supplementary file for figure 4).**

**
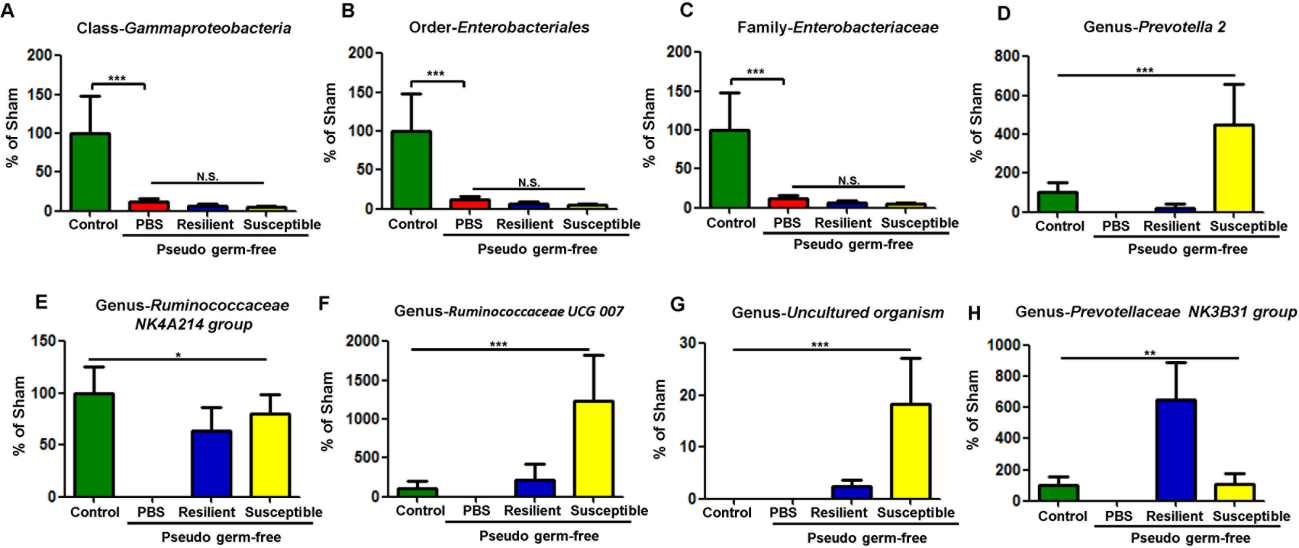
**

**Figure S5. Effects of fecal bacteria transplant on levels of gut microbiota in pseudo-germ-free mice.** (A): Class *Gammaproteobacteria* (F_3,24_ = 6.938_,_ *P* = 0.002). (B): Order *Enterobacteriales* (F_3,24_ = 3.74_,_ *P* = 0.025). (C): Family *Enterobacteriaceae* (F_3,24_ = 3.74_,_ *P* = 0.025). (D): Genus *Uncultured Bacteroidales Bacterium* (F_3,24_ = 5.237_,_ *P* = 0.006). (E): Genus *Ruminococcaceae NK4A214 group* (Fisher’s exact test. *P* < 0.05). (F): Genus *Ruminococcaceae UCG 007* (Fisher’s exact test. *P* < 0.01). (G: Genus *Uncultured organism* (Fisher’s exact test. *P* < 0.001). (H): Genus *Prevotellaceae NK3B31 group* (Fisher’s exact test. *P* < 0.01). **P* < 0.05, ***P* < 0.01 or ****P* < 0.001. Data are shown as mean ± S.E.M. (n = 7). N.S.: not significant.
